# Food-Web Structure of Seagrass Communities across Different Spatial Scales and Human Impacts

**DOI:** 10.1371/journal.pone.0022591

**Published:** 2011-07-21

**Authors:** Marta Coll, Allison Schmidt, Tamara Romanuk, Heike K. Lotze

**Affiliations:** 1 Department of Biology, Dalhousie University, Halifax, Nova Scotia, Canada; 2 Institut de Ciencies del Mar (ICM-CSIC), Barcelona, Spain; National Oceanic and Atmospheric Administration/National Marine Fisheries Service/Southwest Fisheries Science Center, United States of America

## Abstract

Seagrass beds provide important habitat for a wide range of marine species but are threatened by multiple human impacts in coastal waters. Although seagrass communities have been well-studied in the field, a quantification of their food-web structure and functioning, and how these change across space and human impacts has been lacking. Motivated by extensive field surveys and literature information, we analyzed the structural features of food webs associated with *Zostera marina* across 16 study sites in 3 provinces in Atlantic Canada. Our goals were to (i) quantify differences in food-web structure across local and regional scales and human impacts, (ii) assess the robustness of seagrass webs to simulated species loss, and (iii) compare food-web structure in temperate Atlantic seagrass beds with those of other aquatic ecosystems. We constructed individual food webs for each study site and cumulative webs for each province and the entire region based on presence/absence of species, and calculated 16 structural properties for each web. Our results indicate that food-web structure was similar among low impact sites across regions. With increasing human impacts associated with eutrophication, however, food-web structure show evidence of degradation as indicated by fewer trophic groups, lower maximum trophic level of the highest top predator, fewer trophic links connecting top to basal species, higher fractions of herbivores and intermediate consumers, and higher number of prey per species. These structural changes translate into functional changes with impacted sites being less robust to simulated species loss. Temperate Atlantic seagrass webs are similar to a tropical seagrass web, yet differed from other aquatic webs, suggesting consistent food-web characteristics across seagrass ecosystems in different regions. Our study illustrates that food-web structure and functioning of seagrass habitats change with human impacts and that the spatial scale of food-web analysis is critical for determining results.

## Introduction

Seagrasses form extensive underwater meadows that support diverse and complex communities, occur on all continents except Antarctica [1], [2], and are valued as one of the most important marine ecosystems [3] because they provide essential functions and services [1], [4], [5]. Despite this, seagrass habitats around the world are also among the most human impacted marine ecosystems [6].

Eelgrass, *Zostera marina,* is the most widely distributed seagrass species in the world and dominates coastal and estuarine habitats of the temperate North Atlantic, including Atlantic Canada [5], [7]. Globally, eelgrass beds are subject to natural and anthropogenic impacts that have caused declines, and in some cases, local extinction [6], [8]. However, they generally receive little protection even if they are key habitats. In Canada, although eelgrass has been recently listed as an ecologically significant species [9], no specific legal protection exists for seagrass communities and very few beds are included in marine protected areas [7].

Among the multiple anthropogenic impacts on seagrass beds, eutrophication has been identified as a major cause for seagrass declines around the world [4], [6], [10]. Nutrient loading increases the concentration of nitrogen and phosphorous in the water thereby enhancing the growth of annual micro- and macroalgae [11]. The increase in phytoplankton, epiphytic, and free-floating macroalgae reduces the amount of light reaching seagrass for photosynthesis and growth, while the decomposition of dead algal matter enhances oxygen depletion and the development of anoxic sediments [4], [12]. The result is a reduction in above (blades, sheaths, inflorescences) and below (rhizomes, rootlets) ground seagrass production [4]. For example, in Waquoit Bay (Cape Cod, Massachusetts) seagrass beds have practically disappeared over the past century due to nutrient loading [13]. Although less severe, signs of eutrophication have also been observed in seagrass beds in Atlantic Canada [12], [14].

Changes in seagrass beds can alter the structure and function of associated ecosystems and the goods and services they provide to humans [12], [15], [16]. Changes in trophic relations in seagrass food webs due to eutrophication have been studied using stable isotopes, trophic guilds, gut contents, and trophic models (e.g. [15], [16], [17], [18]). These studies found important changes in the trophic positions of organisms and trophic flows subjected to high levels of nutrients. However, the overall changes in food-web structure have not been fully described, and studies available are limited in spatial coverage. Since oceanic nutrients can vary over large spatial scales [19] an important next empirical step is to consider how interactions such as those within *Zostera marina* food webs could change at larger scales.

To address these gaps, we used a combination of large-scale field surveys and food-web modeling to (i) quantify the main structural features of food webs associated with *Z. marina* across local and regional scales and human impacts in Atlantic Canada, (ii) assess whether structural differences translate into changes in functioning by analyzing the robustness of food webs to simulated species loss, and (iii) compare the structure of seagrass food webs in Atlantic Canada with other aquatic food webs to determine whether seagrass webs have unique and consistent features. For our food-web analysis, we chose a widely-used binary network approach ([20], [21], www.foodwebs.org) due to its simplicity and few required assumptions and parameters. Previous work has shown that binary network models and more complex biomass and trophic flow models deliver comparable results when analyzing structural food-web degradation, suggesting that both approaches capture fundamental information about how food webs are structured and change under human pressures [22].

## Methods

### 1. Study sites

**Figure 1:**
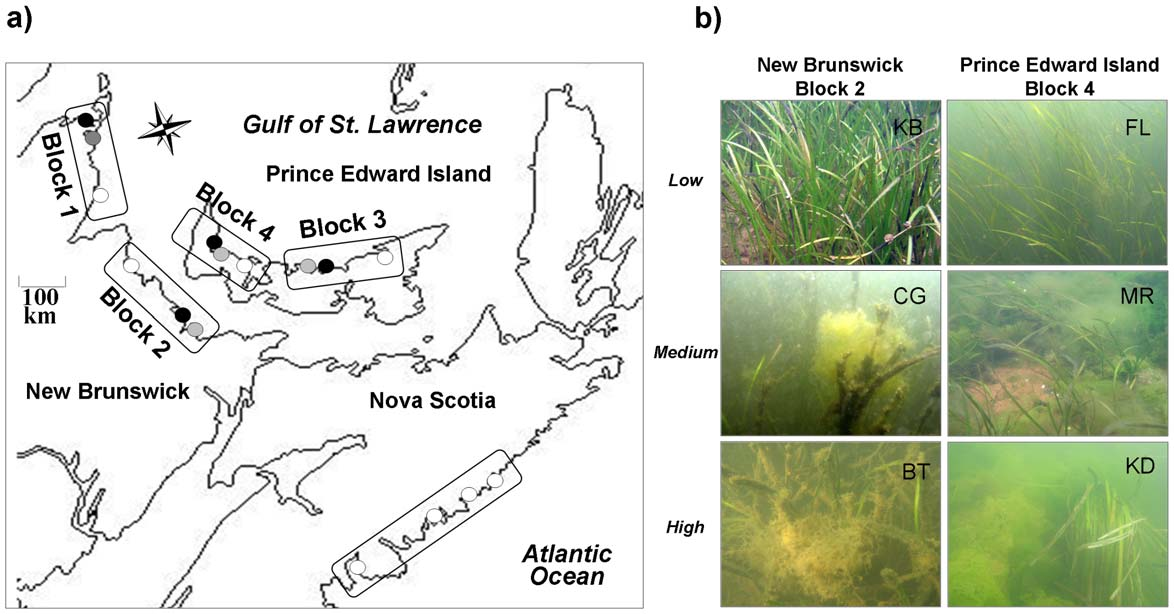
Study area in Atlantic Canada. A) Study sites located in New Brunswick, Prince Edward Island, and Nova Scotia (see Table 1 for details). Study sites are indicated with low (open circles), medium (grey circles), and high (black circles) impact levels. B) Underwater photos of seagrass beds of NB and PEI (Block 2 and 3 in Fig. 1a, respectively).

Our study was conducted in a vast area of eastern Canada (Fig. 1a). We selected twelve sites along the Gulf of St. Lawrence coast of New Brunswick (NB) and Prince Edward Island (PEI), sampled once from 27 July to 8 August 2007, and four sites along the Atlantic coast of Nova Scotia (NS), sampled from 15–20 August 2007 (Fig. 1a, Table 1). In NB and PEI, sites were allocated to a block and arrayed along a gradient of human impacts associated with eutrophication (Low, Medium, High), while in NS all sites exhibited low impact levels (Fig. 1a, Table 1, see below). Each study site was located in a distinct bay or estuary, often separated by barrier islands from open waters of the Gulf of St. Lawrence (NB, PEI) or Atlantic Ocean (NS). Thus, individual sites were influenced by local conditions and relatively independent of each other, but all sites had similar temperature and salinity conditions [14]. Although there is a general lack of comprehensive coastal mapping data in most of Atlantic Canada, some broad estimates of seagrass extent exist documenting ∼20000 ha of eelgrass in NB and 30000 ha in PEI [9]. In addition, the comparison of some historical and more recent data highlights important declines of seagrass beds in several locations [23].

**Table 1:** Study sites by province, block and impact level based on anthropogenic activities (x  =  present).

Site	Code	Location	Impact level	Eutrophication PCA score 1	Fish proces-sing plant	Shellfish aquaculture	Urban sewage, septic systems	Industry	Agriculture
***New Brunswick***									
**Block 1**									
Tabousintac Bay	TB	N 47°22′56, W 64°56′21	Low	−1.27			x		
Baie St. Simon Sud	BS	N 46° 29′70, W 64°40′47	Medium	−1.01		x	x		x^1^
Lameque Bay	LM	N 47°47′44, W 64°40′31	High	0.74	x	x	x	x	x^1^
**Block 2**									
Kouchibouguac National Park	KB	N 46° 50′30, W 64°56′16	Low	−0.87					
Cocagne Bay	CG	N 46°22′01, W 64°36′95	Medium	0.84		x	x	x	x^2^
Bouctouche Bay	BT	N46°29′70, W 64°47′47	High	2.20		x	x	x	x^2^
***Prince Edward Island***									
**Block 3**									
Stanley trout Estuary	ST	N 46°28′47, W 63°27′84	Low	−1.28		x	x		x^3^
Midgell Estuary	MD	N 46°25′01, W 62°37′60	Medium	0.38		x	x		x^3^
Southwest Estuary	SW	N 46°28′75, W 63°30′38	High	1.85		x	x	x	x^4^
**Block 4**									
Freeland Estuary	FL	N 46°41′29, W 63°56′40	Low	−1.10		x			x^5^
Mill River Estuary	MR	N 46°45′91, W 64°04′72	Medium	−0.21		x	x		x^6^
Kildare Estuary	KD	N 46°49′96, W 64°02′97	High	3.24	x		x	x	x^7^
***Nova Scotia***									
Taylor's Head Provincial Park	TH	N 44°49′26, W 62°34′32	Low	−0.93					
False Passage	FP	N 44°44′37, W 62°47′45	Low	−1.42					
Musquodoboit Harbour	MH	N 44°42′46, W 63°04′48	Low	0.19			x		x^8^
Franks George Island	FG	N 44°35′68, W 63°53′73	Low	−1.34					

Eutrophication level is indicated by the scores of PC axis 1 based on PCA of C/N ratios in seagrass tissue, chlorophyll concentrations in the water column, epiphytic and benthic annual algae biomass (see text for detail and Methods S1 for data).

1 - Peat Mining <5 km from site; 2 - agriculture <10 km upstream; 3 −85% agriculture within a 2 km radius of the site but still heavily forested 4–6 km upriver; 4 −95% agriculture within a 2 km radius of the site no forest left along the banks of the river; 5 −5% agriculture within a 2 km radius of the site, area still heavily forested; 6 −80% agriculture within a 2 km radius; 7 −45% agriculture within 2 km of site, heavy agriculture up river; 8 - Industrial forestry 3–4 km upriver.

Impact levels were chosen based on previous sampling in NB [12] and nutrient concentrations in rivers and estuaries in PEI (Surface water quality database; Government of Prince Edward Island; Department of Environment, Energy and Forestry; http://www.gov.pe.ca/eef/). They were then confirmed using carbon to nitrogen (C/N) ratios in seagrass tissue, chlorophyll-a concentrations in the water column (µg l^−1^), and biomass of annual epiphytic and benthic macroalgae (g m^−2^) collected during field sampling. Although there was variability among study sites and regions, there was a general decrease in C/N and increase in chlorophyll-a as well as an increase in annual algae biomass along the impact gradient (Methods S1). Results of a Principal Component Analysis (scores of PC axis 1, explaining >50% or variance, Methods S1) indicated a clear impact gradient within each block. These gradients were also corroborated by the presence of human activities related to nutrient loading and habitat alteration (Table 1) and underwater images (Fig. 1b). Other human impacts, particularly exploitation, occurred throughout the region and no site was located in a marine protected area.

### 2. Sampling procedure and data collection

We used different sampling techniques to collect all major biotic components of seagrass communities. Transects (50 m long, 4 m wide) were deployed parallel to the shore inside (10 m from any edge) the seagrass bed to visually census highly mobile macrofauna during day and night high tides. The transect depth was 1.2–1.8 m in NB and PEI, and 2–4 m in NS where the seagrass beds occur at greater depth. During the day, we identified all sessile benthic and epiphytic fauna and flora as well as small, slow-moving, and cryptic macrofauna using 11 quadrats (50×50cm) placed every 5 m along the transect line. We excluded highly mobile species identified with visual census from the quadrat results. In 3 quadrats (at 0, 25 and 50 m along the transect line) we collected a sediment core (0.2 m diameter; 0.2 m deep) to sample the infauna. All species were identified to the lowest possible taxonomic level (Methods S2). Relevant permits for our observational and field studies were obtained from national institutions (Parks Canada and the Department of Fisheries and Oceans).

### 3. Food-web networks and properties

**Figure 2:**
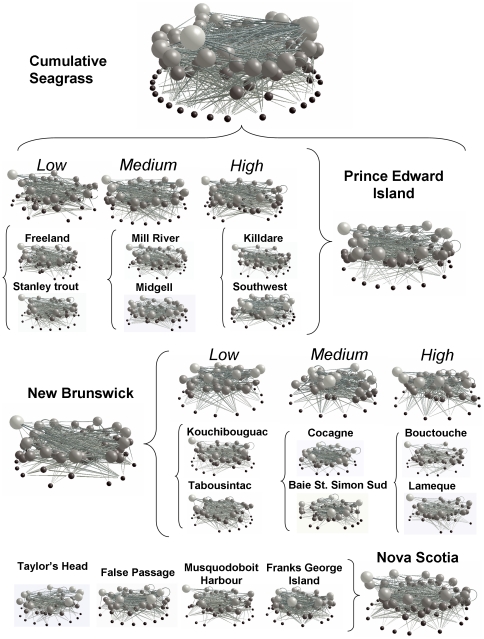
Visualization of the seagrass food webs by site and aggregated by eutrophication level and region. Different coloured dots represent trophic groups from different trophic levels with black  =  primary producers, dark to light grey  =  secondary producers, and the lightest grey being top predators. The grey links represent feeding links.

Based on all species identified during field sampling, including primary producers, benthic and pelagic invertebrates, fishes, and other vertebrates, we constructed (1) individual food webs for each study site (n = 16). Data from different sites were then aggregated into cumulative food webs for (2) each region and impact level (NB-low, NB-medium, NB-high, PEI-low, PEI-medium, PEI-high), (3) each region (NB, PEI, NS), and (4) the overall seagrass community in Atlantic Canada (Fig. 2). These food webs were then used to test for differences in food-web structure across impact levels and regions (1, 2), and across different spatial scales of data accumulation (2, 3, 4). Such spatial accumulation is common practice in food-web construction to best represent all species and interactions possible in a region (e.g. [21]), yet may unintentionally omit site-specific food-web differences.

We identified a total of 86 species plus 26 genera: 25 species plus 11 genera of primary producers, 41 species and 14 genera of invertebrates, and 20 species of vertebrates (Methods S2). Epiphytes on seagrass consisted of red (e.g. *Polysiphonia* spp.), brown (e.g. *Scytosiphon* spp.) and green algae (e.g. *Ulothrix speciosa*), polychaetes (*Spirorbis* spp.), and bryozoans (e.g. *Electra pilosa*, *Membranipora membranacea*). We recorded four invasive species: green crab (*Carcinus maenas*), oyster drill (*Urosalpinx cinerea*), green fleece (*Codium fragile* spp. *tomentosoides*), and sea mat (*M. membranacea*). Five marine mammal and 16 bird species were added to the food webs based on our own and published field observations and distribution ranges (Methods S2).

For the food-web construction, we obtained species- and region-specific diet information from the literature (Methods S3). The information on trophic links was used to create a matrix of prey-predator relationships. When species-specific information was not available, taxa were assigned to trophic groups composed of similar species according to ontogenetic stages (e.g. eggs, larvae, and adults) and ecological characteristics (e.g. feeding, habitat, and mortality) as in the groups of generic macroalgae, zooplankton, or demersal fish (Methods S4). Two detrital groups, suspended detritus and deposited organic matter, were also used and a group to account for import diets into the system. Efforts were made to sample and include as many organisms in the food web as possible with the aim of preventing bias towards higher trophic level organisms in our models. However, smaller organisms such as infauna are more difficult to sample and there is less information regarding feeding behavior, thus our food web represents the higher trophic level organisms with better detail. However, this limitation is consistent to all our models and does not impact our results which follow a comparative approach.

Using a binary network approach (www.foodwebs.org) we calculated 16 structural food-web properties (Table 2) based on previous work [20], [21]. We used published literature and previous studies on food-web degradation [12], [21], [22], [24], [25], [26] to outline expected trends of each food-web property with increasing eutrophication and degradation in seagrass systems (Table 2). Food-web images were produced with FoodWeb3D, written by R.J. Williams, Pacific Ecoinformatics and Computational Ecology Lab.

**Table 2:** Food-web properties used to characterize food-web structure and the predicted trend with increasing degradation based on the literature [21], [22], [24], [25], [26].

		Food-web properties	Description	Predicted trend
**1**	**S**	**Trophic groups**	Species or groups of species used to build the food-web models	Decrease
**2**	**L/S**	**Linkage density**	All trophic links in the web (L) divided by S (number of species or ecological groups)	Decrease
**3**	**C**	**Connectance**	Proportion of actual trophic links to all possible links (L/S^2^), 0 = no species preys on any species, 1 = every species preys on every other species including itself	Decrease
**4**	**GenSD**	**Generality**	Number of prey items per species and standard deviation	Increase
**5**	**VulSD**	**Vulnerability**	Number of predators per species and standard deviation	Decrease
**6**	**%T**	**Fraction of top predators**	Fraction of species with prey but no predators	Decrease
**7**	**%I**	**Fraction of intermediate predators**	Fraction of species with both prey and predators	Increase
**8**	**%B**	**Fraction of basal species**	Fraction of species with predators but no prey	Increase
**9**	**%H**	**Fraction of herbivore species**	Fraction of species that feed on primary producers	Increase
**10**	**SWTL**	**Mean trophic level of the community**	Short-weighted trophic level (SWTL) or the average of prey trophic level	Decrease
**11**	**MaxTL**	**Maximum trophic level**	Short-weighted maximum trophic level of the top predator in the system	Decrease
**12**	**%Omn**	**Fraction of omnivorism**	Fraction of species that feed directly on more than one trophic level and have food chains of different lengths	Decrease
**13**	**%Can**	**Fraction of cannibalism**	Fraction of species that feed directly on their own species	Decrease
**14**	**%Loop**	**Fraction in loop**	Fraction of species involved in looping by appearing in a food chain twice	Decrease
**15**	**ChLen**	**Mean short-weighted chain length**	Mean number of links in every possible food chain or sequence of links connecting top to basal species	Decrease
**16**	**Path**	**Trophic path length**	Characteristic path length or the mean shortest path length between species pairs	Decrease

In order to place our results into a wider context, we evaluated how our webs compared to 14 other aquatic food webs located worldwide that were previously built using similar methodology which included marine (e.g. Caribbean Sea), estuarine (e.g. Chesapeake Bay), lotic (e.g. Canton Creek), and lentic (e.g. Skipwith Pond, Little Rock Lake) ecosystems [20]. From the estuarine group, we separated a seagrass-dominated tropical estuary (Saint Mark's estuary, [20], [27]) that differed from non-seagrass dominated estuaries. We also created a new group for our temperate seagrass food webs (NS, NB, PEI, Atlantic) based on our own data. For each of the 18 food webs in 6 groups, we extracted eleven common food-web properties for structural comparison.

### 4. Statistical analysis

Principal Component Analysis (PCA) of trophic groups used to develop food webs by study site was used to examine each of the 16 individual food webs. Data were first √-transformed to avoid over-domination of very common groups [28].

In addition, to test for differences across blocks (4 levels, Fig. 1a) within regions (2 levels: NB, PEI) we used site-specific food webs in a two-way nested analysis. Because the block effect was not significant, we then used a two-way fully crossed analysis comparing regions (NB, PEI) and impact levels (Low, Medium, High). We also used a one-way analysis on the low impact sites only comparing regions (NB, PEI, NS) and to test for large-scale differences in common food-web properties among the six groups of aquatic ecosystems (marine, estuarine, lotic, lentic, seagrass-tropical and seagrass-temperate). All analyses were performed using multivariate permutational analysis of variance (PERMANOVA) on the Euclidean distance matrix of food-web properties, which allows for the analysis of more complex designs (multiple factors and their interaction) without the constraints of multivariate normality, homoscedasticity, and having a greater number of variables than sampling units of traditional MANOVA. This method calculates a *pseudo-F* statistic directly analogous to the traditional *F*-statistic for multifactorial univariate ANOVA models but uses permutation procedures to obtain p-values for each term in the model [29]. If significant differences occurred, a univariate PERMANOVA was conducted for individual food-web properties. We selected the unrestricted permutation of raw data procedure for p-value calculation because it generally has a Type I error rate close to α for multivariate models and is an exact test for univariate models. It is also the best option for small sample sizes (<4 replicates, [29]). We used a significance level of α = 0.05 yet mention levels up to α  = 0.1 because expected changes in food-web properties are generally small yet may still be biologically relevant.

Secondly, we used non-metric multi-dimensional scaling (MDS) and cluster analysis based on Euclidean distances to visualize differences among i) individual food webs with low impact levels, ii) cumulative food webs across regions and impact levels (NB-low, NB-medium, NB-high, PEI-low, PEI-medium, PEI-high) and across regions (NB, PEI, NS, Atlantic), and iii) all aquatic ecosystems. The MDS analysis used random starting configurations and 1000 runs with real data. A two-dimensional representation was accepted as a good depiction of the data if the stress index was ≤0.1 [28]. We used SIMPER analysis [30] to identify the network properties that contributed to ≥10% of the differences among data points. All analyses (PCA, MDS, PERMANOVA, and SIMPER) were performed using PRIMER with PERMANOVA+ (v. 6, PRIMER-E Ltd., Plymouth, UK).

For each test, we first assessed skewness and individual correlations between food-web properties by constructing a draftsman plot (matrix of plots of each food-web property against the other), and examining the resulting Spearman rank correlations. Properties that were skewed to the right or left were log(x) or reverse (log(c-x), where c > max x) transformed, respectively. We removed one of each pair of properties that were significantly correlated (ρ≥0.85); thereby reducing redundancy in and dimensionality of the data. Because the properties represented different measures (%, counts, etc.), they were all normalized prior to the construction of a Euclidean distance matrix [31].

### 5. Extinction simulations

To examine whether changes in food-web structure translated into changes in functioning, we explored the potential effect of simulated species removal to trigger cascades of secondary extinctions [32]. A secondary extinction is defined as a non-basal species losing all of its prey and a cannibalistic species losing all of its prey items except itself. To perform this analysis we used the cumulative food webs by region and impact level (NB-low, NB-medium, NB-high, PEI-low, PEI-medium and PEI-high, NS-low) and by regions (NB, PEI, NS, Atlantic). Species losses were simulated sequentially by removing 1) the least connected species, 2) the most connected species, and 3) species randomly chosen from 1000 random removal sequences initiated for each food web. Basal species, which are those species with predators but no prey (see Methods S2 for specific species), were protected from being removed. Extinction analyses were performed using the software WebProg-Node Knockouts [32].

## Results

### 1. Species and trophic groups

**Figure 3:**
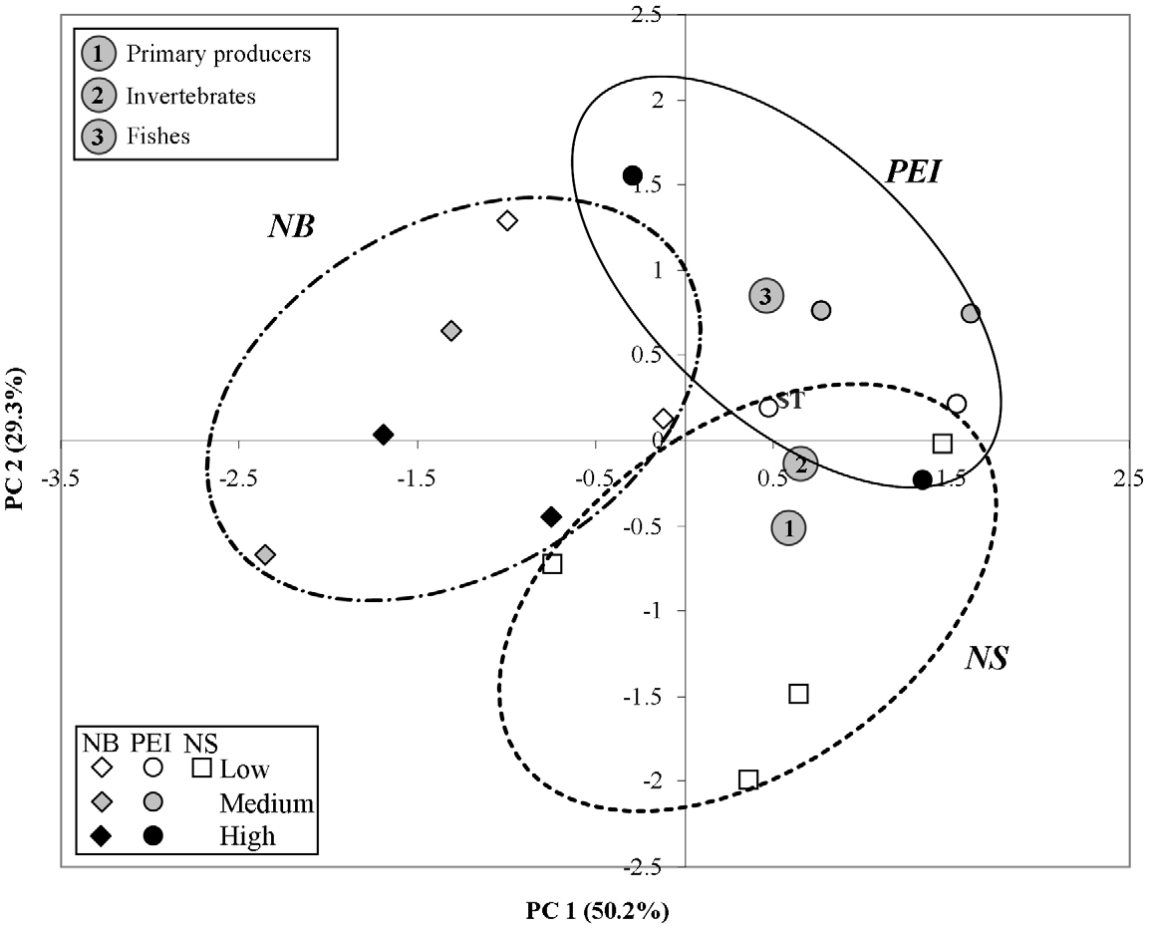
Principle components analysis of trophic groups used to develop food webs by study site. Each point represents a study site within a Province (NB  =  New Brunswick, PEI  =  Prince Edward Island, NS  =  Nova Scotia) and impact level (Low, Medium, High). The larger circles indicate sites by region, and 1, 2 and 3 indicate the position of the main characterizing trophic groups (primary producers, invertebrates and fishes) along the two principle components.

The cumulative Atlantic seagrass food web had 107 trophic groups including 25 primary producers and detritus components, 36 invertebrate, 45 vertebrate groups, and an “import” group to account for import diets into the system (Methods S4). Functional group richness for site-specific webs had a mean of 62 (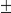
4.4 SD) groups (Table 3). A higher number of trophic groups were necessary to describe PEI food webs, followed by NS and NB (Fig. 3). PEI webs were characterized by a higher number of fish groups and fewer primary producers and invertebrates. NS webs had the highest number of primary producers and NB webs had fewer groups of fishes, invertebrates, and primary producers.

**Table 3:** Food-web properties (see Table 2 for definitions) for each site (see Table 1 for site abbreviations) grouped by region (New Brunswick, Prince Edward Island, Nova Scotia) and impact level due to eutrophication (low, medium, high).

Food-web properties	a) New Brunswick						b) Prince Edward Island						c) Nova Scotia			
	Block 1			Block 2			Block 3			Block 4						
	TB	BS	LM	KB	CG	BC	ST	MD	SW	FL	MR	KD	FP	FG	MH	TH
	Low	Medium	High	Low	Medium	High	Low	Medium	High	Low	Medium	High	Low	Low	Low	Low
**S**	59	53	59	61	57	56	63	65	66	68	68	62	60	67	62	63
**L/S**	11.68	12.11	12.42	11.38	11.63	11.55	11.73	11.65	11.59	11.21	11.25	12.18	12.92	12.16	11.65	11.51
**C**	0.20	0.23	0.21	0.19	0.20	0.21	0.19	0.18	0.18	0.17	0.17	0.20	0.22	0.18	0.19	0.18
**GenSD**	0.96	1.03	0.97	0.96	0.98	0.99	1.02	1.01	1.01	0.95	1.04	0.99	0.94	0.96	1.04	1.06
**VulSD**	1.81	2.12	2.01	1.93	2.07	1.99	1.85	1.88	1.90	1.91	1.82	1.87	1.79	1.54	1.91	1.85
**%T**	0.17	0.17	0.15	0.15	0.16	0.18	0.14	0.15	0.15	0.16	0.13	0.15	0.15	0.13	0.16	0.14
**%I**	0.59	0.59	0.63	0.62	0.61	0.61	0.62	0.63	0.61	0.63	0.65	0.65	0.63	0.64	0.58	0.62
**%B**	0.24	0.25	0.22	0.23	0.23	0.21	0.24	0.22	0.24	0.21	0.22	0.21	0.22	0.22	0.26	0.24
**%H**	0.07	0.08	0.05	0.07	0.09	0.11	0.08	0.08	0.08	0.09	0.09	0.10	0.07	0.08	0.07	0.02
**SWTL**	1.83	1.77	1.85	1.86	1.83	1.86	1.85	1.88	1.83	1.89	1.90	1.87	1.86	1.86	1.82	1.97
**MaxTL**	3.58	2.72	3.58	3.58	2.91	3.58	3.58	3.14	2.93	3.58	3.58	3.58	2.99	3.11	3.58	3.41
**%Omn**	0.73	0.70	0.75	0.74	0.72	0.73	0.73	0.74	0.73	0.77	0.74	0.73	0.73	0.75	0.71	0.71
**%Can**	0.17	0.13	0.19	0.15	0.16	0.14	0.16	0.14	0.15	0.15	0.15	0.15	0.18	0.15	0.15	0.05
**%Loop**	0.10	0.04	0.12	0.03	0.07	0.04	0.05	0.08	0.05	0.06	0.07	0.07	0.13	0.08	0.08	0.46
**ChLen**	1.90	1.94	1.97	1.95	1.95	1.98	1.95	1.95	1.92	1.97	1.96	1.97	1.97	1.96	1.94	2.05
**Path**	2.03	2.06	2.04	2.04	2.00	2.02	2.07	2.04	2.08	2.08	2.09	2.01	1.94	2.03	2.11	2.06

### 2. Individual food webs across regions and impact gradient

**Figure 4:**
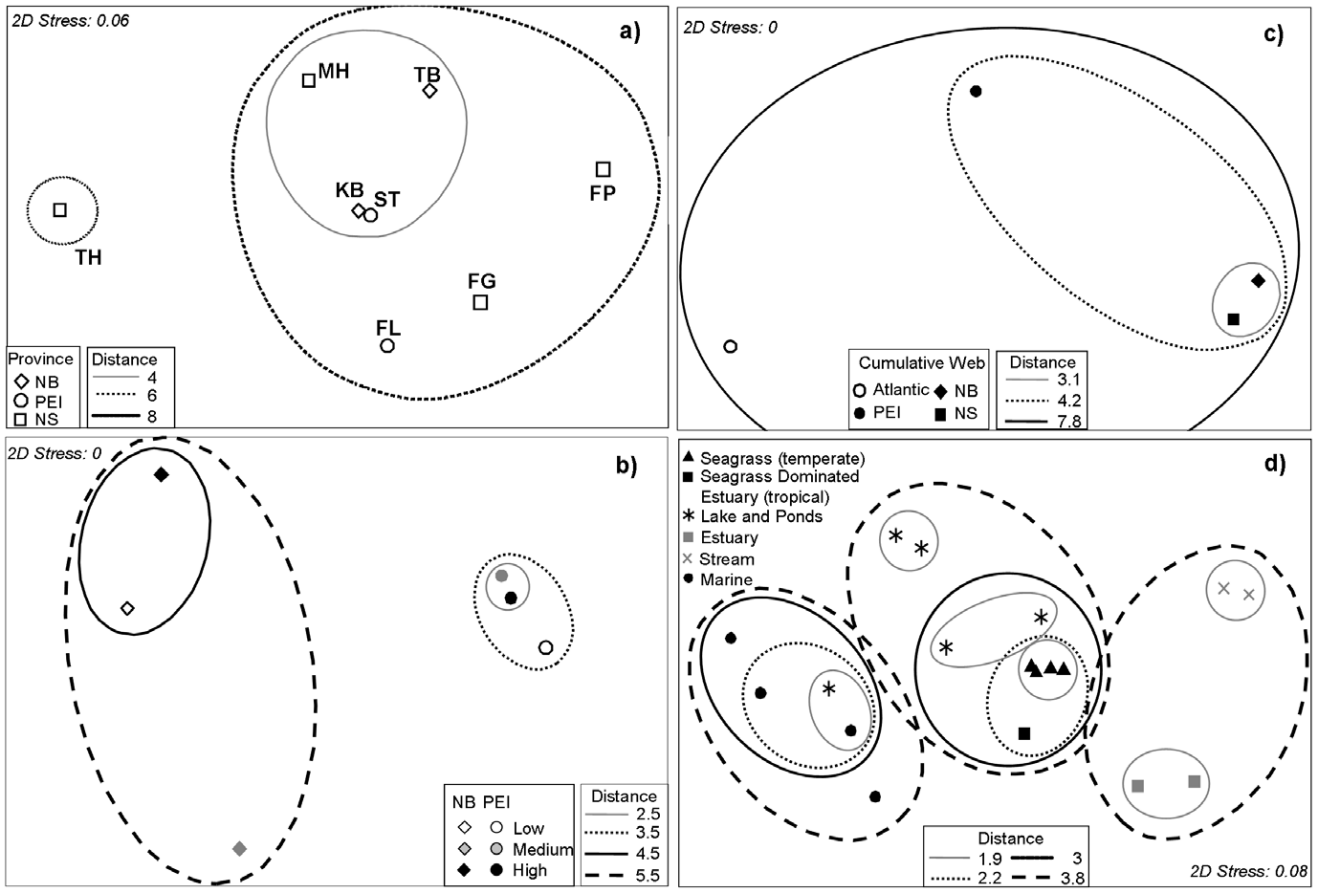
Multidimensional scaling (MDS) overlaid with Euclidean distances from cluster analysis of food-web properties. A) food webs from low impacted sites (Table 3), b) cumulative food webs by region and impact (NB-low, NB-medium, NB-high, PEI-low, PEI-medium, PEI-high, Table 4), c) cumulative food webs by region (NB, PEI, NS) and the Atlantic seagrass web (Table 4), and d) 14 aquatic food webs from Dunne et al. [20] and four cumulative food webs from this study (NS, NB, PEI, Atlantic) depicted as temperate seagrass webs.

**Figure 5:**
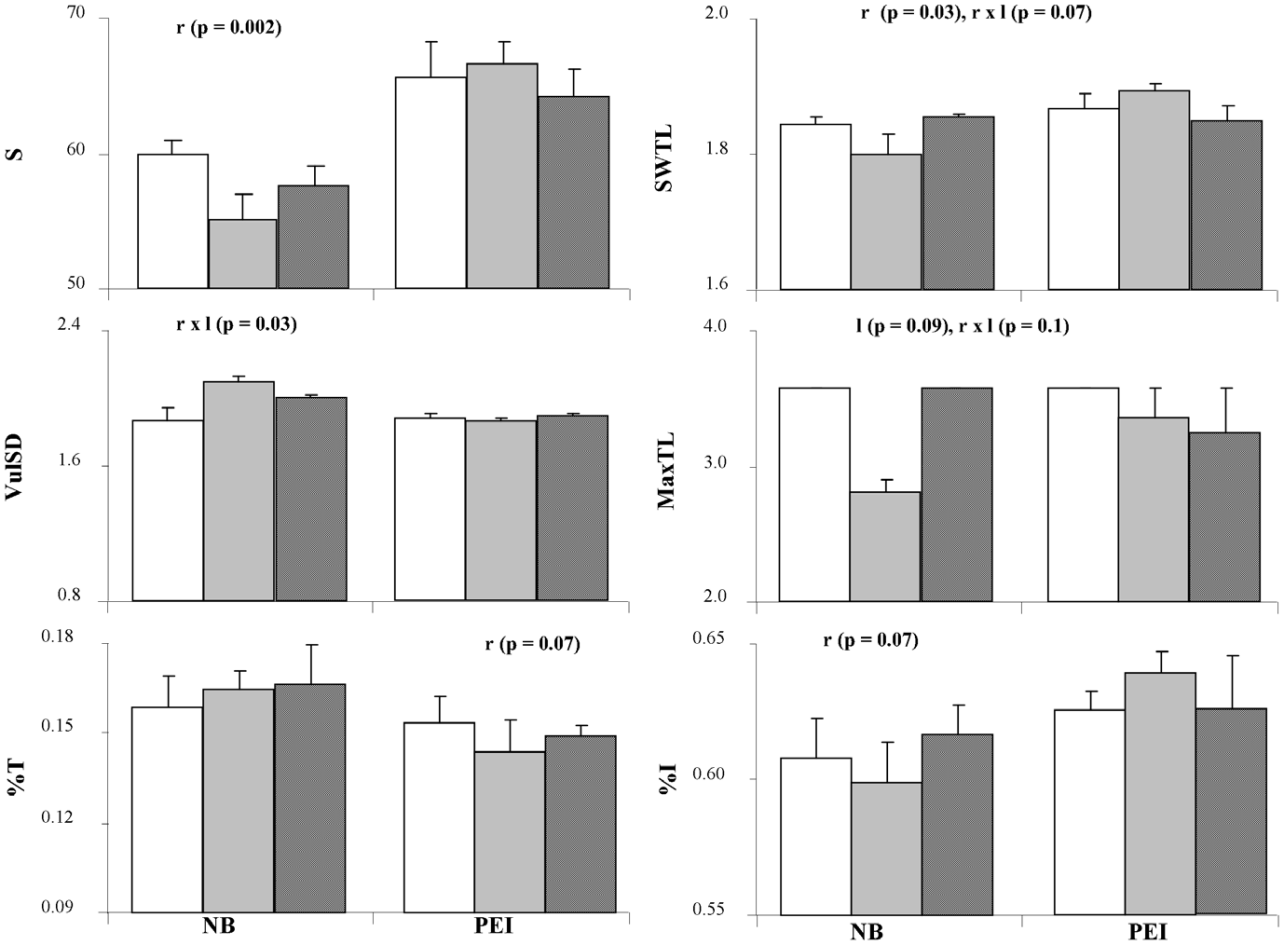
Food-web properties (**Table 3**) with differences between regions, impact level, and their interaction using two-way protected permutational ANOVA. Significant (α = 0.05) or biological important (α = 0.1) differences between factors are indicated by regions (r), level (l), and their interaction (r x l). Data are means (± SE; n = 2); see Table 2 for abbreviations of food-web properties. Regions  =  NB or PEI. Impact level  =  Low: white, Medium: grey, High: hatched bars.

We found no differences in food-web properties among all study sites with low impact levels in NB, PEI and NS (PERMANOVA, pseudo-F_2,5_ = 0.77, p = 0.75) and no clear regional grouping in the MDS ordination (Fig. 4a). However, when we tested for the effect of region and impact level among NB and PEI sites, we found a significant effect of region (pseudo-F_1,6_ = 2.49, p = 0.02) but not of impact level or their interaction (p>0.50). Univariate PERMANOVA on each food-web property revealed higher number of trophic groups (S) and short-weighted trophic level (SWTL) in PEI than NB (Fig. 5), and a trend towards a higher fraction of intermediate (%I) and lower top (%T) predators in PEI than NB. Moreover, within each region there was a tendency towards decreasing S, increasing %I and decreasing %T (except NB) from low to high impacted sites (as predicted in Table 2), but with considerable variability (Table 3). Interactions between region and impact level occurred for the vulnerability (VulSD) (Fig. 5). SWTL and MaxTL tended to decrease (as predicted in Table 2) from low to high impact in PEI, although his difference was not significant. VulSD did not follow the predicted decrease in both regions.

Because of the large number of food-web properties involved, the low number of replicates, and high variability in response among sites, the lack of strong significant effects of the impact gradient is not surprising. Overall changes in individual food-web properties were small (Table 3), as expected given the number of species in the webs and that changes relate to presence/absence only. However, overall, 75% of food-web properties in PEI and and 43% in NB followed a trend towards higher degradation with increasing impact (Table 3), as predicted in Table 2. In NB, S, SWTL, MaxTL, and the trophic path length (Path) decreased from low to high eutrophication, while %I, the fraction of herbivore species (%H), and the generality (or number of prey per species, GenSD) increased. In PEI, S, %T, SWTL, MaxTL, the mean short-weighted chain length (ChLen), Path, and the fractions of omnivory (%Omn) and cannibalism (%Can) decreased as well, while %I, %H, the fraction of basal species (%B), and GenSD increased from low to high eutrophication.

### 3. Cumulative food webs across regions and impact gradient

**Figure 6:**
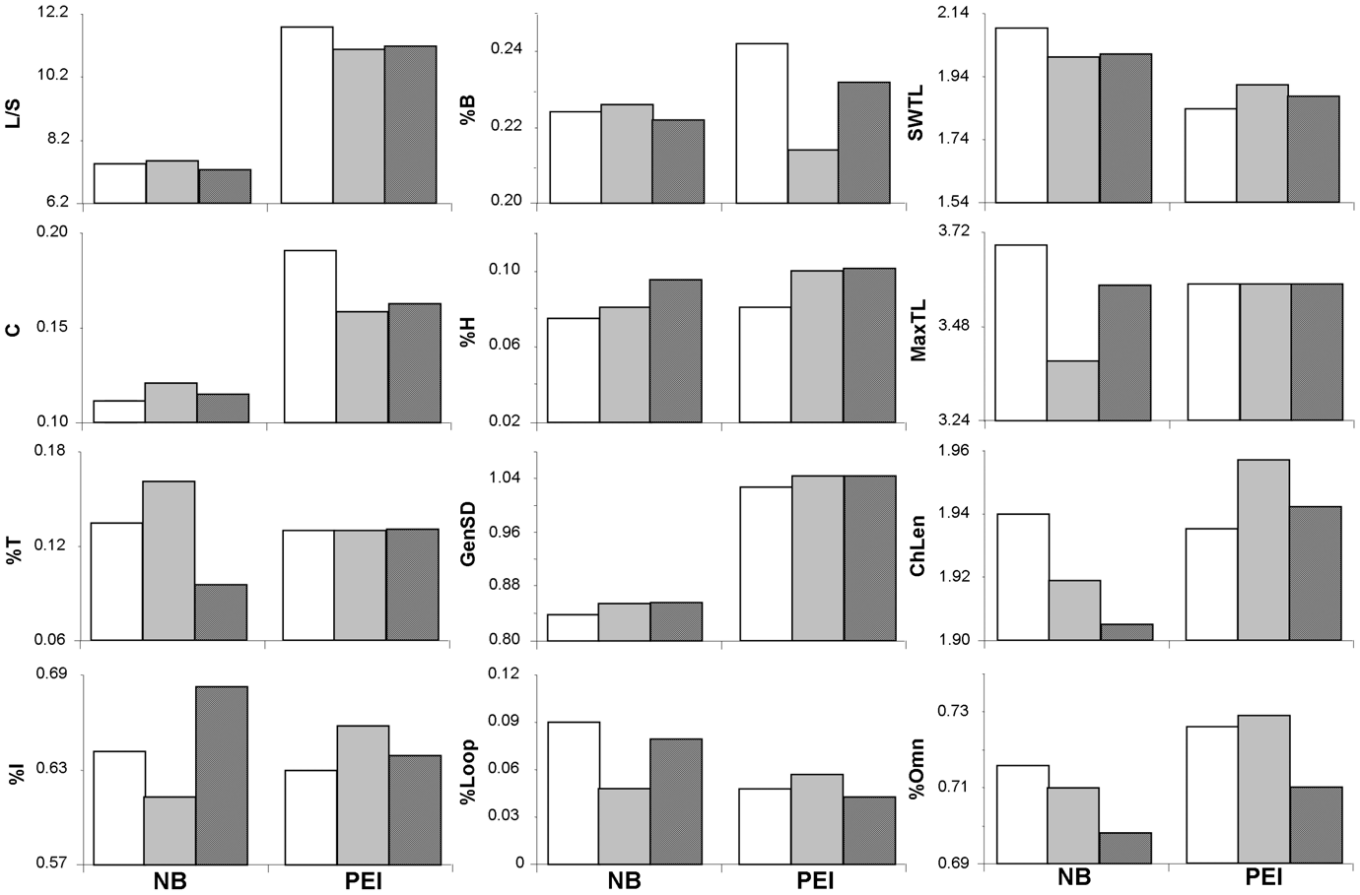
Food-web properties (**Table 4**) identified by SIMPER analysis that contributed to ≥10% of the overall difference between cumulative region by impact webs. Regional food webs are NB-low, NB-medium, NB-high, PEI-low, PEI-medium, PEI-high, with low  =  white, medium  =  grey, and high  =  hatched bars (n = 1). See Table 2 for abbreviations of food-web properties.

MDS ordination of cumulative food webs showed a clear distinction between regions (NB, PEI) (Fig. 4b) and illustrated regional differences across impact levels. In NB, the low and high levels were grouped, whereas in PEI, the medium and high levels were more closely grouped. The PEI food webs were generally more similar to the NB food webs. SIMPER analysis (Fig. 6) indicated that 12 of 16 food-web properties contributed to ≥10% of the differences in at least one of the pair-wise comparisons among cumulative food webs for region and impact level. Notably, PEI had much higher L/S, C, and GenSD than NB, and both L/S and C declined and GenSD increased with increasing eutrophication (as expected in Table 2), at least in PEI. There was a trend of declining %T in NB, and increasing %I and %H (but not %B) with increasing impact in both NB and PEI (as expected in Table 2). In NB, there was also a decline in SWTL, MaxTL, ChLen, and %Omn from low to high, while responses in PEI were more variable. Again, several parameters showed non-linear responses and there was considerable variability. However, among cumulative food webs, 69% of food-web properties in NB and 50% in PEI followed the expected trend of degradation with increasing eutrophication (Table 2, 4).

**Figure 7:**
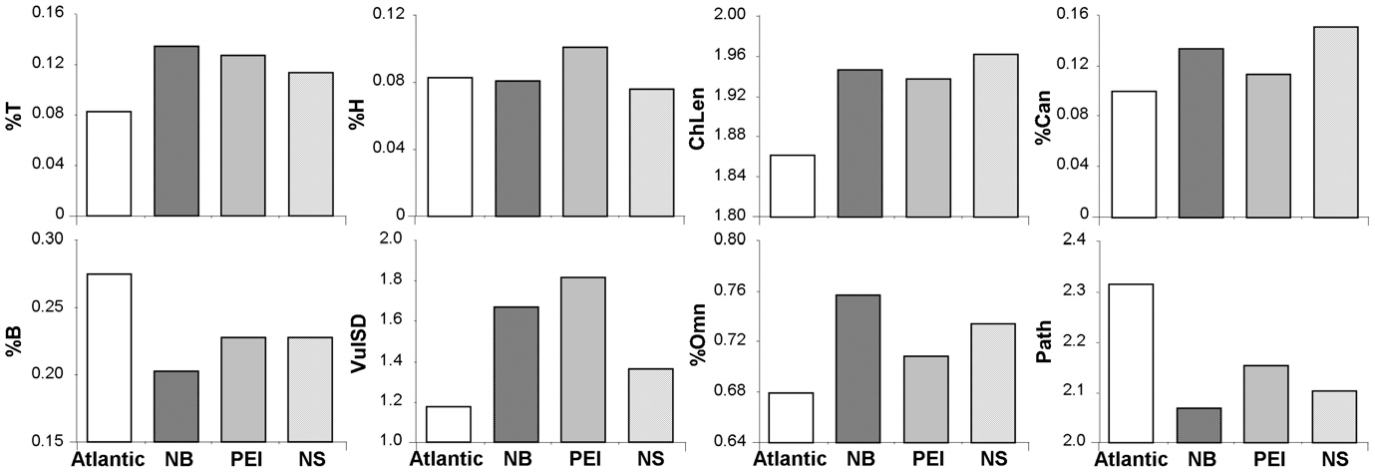
Food-web properties (**Table 4**) identified by SIMPER analysis that contributed to ≥10% of the overall difference between cumulative regional webs. Cumulative regional webs are NB, PEI, NS, Atlantic. See Table 2 for abbreviations of food-web properties.

To test the effect of increasing spatial aggregation on food-web structure, we compared the cumulative regional (NS, NB, PEI) and overall Atlantic food webs (Table 4, Fig. 4c). Here, NS and NB were most similar, PEI was in an intermediate position, and all regional webs were quite different from the overall Atlantic web. SIMPER identified eight food-web properties as contributing the greatest to these differences (Fig. 7). Overall, the Atlantic web showed lower %T and higher %B than the regional webs. Also, ChLen, %Can, VulSD, and %Omn were lower and Path higher in the Atlantic compared to regional webs. Only %H was similar between the Atlantic and regional webs with the exception of higher %H in PEI. This analysis illustrates that the spatial scale at which food-web properties are studied (covering the overall region or different sub-regions) affect food-web topology and the conclusions drawn from resulting analyses.

**Table 4:** Food-web properties (see Table 2 for definitions) of the cumulative food webs for a) each region and impact level, b) each region, and c) the region of Atlantic Canada.

Food-web properties	a) Cumulative food web by province and impact level						b) Cumulative food web by province			c) Atlantic Canada
	NB low	NB medium	NB high	PEI low	PEI medium	PEI high	NB	PEI	NS	
**S**	67	62	63	62	70	69	74	79	79	107
**L/S**	7.43	7.53	7.25	11.77	11.07	11.17	11.28	10.61	11.20	9.28
**C**	0.11	0.12	0.12	0.19	0.16	0.16	0.15	0.13	0.14	0.09
**%T**	0.13	0.16	0.10	0.13	0.13	0.13	0.14	0.13	0.11	0.08
**%I**	0.64	0.61	0.68	0.63	0.66	0.64	0.66	0.65	0.66	0.64
**%B**	0.22	0.23	0.22	0.24	0.21	0.23	0.20	0.23	0.23	0.28
**%H**	0.08	0.08	0.10	0.08	0.10	0.10	0.08	0.10	0.08	0.08
**GenSD**	0.84	0.86	0.86	1.03	1.04	1.04	0.99	1.06	0.99	1.11
**VulSD**	1.54	1.77	1.55	1.79	1.84	1.83	1.67	1.82	1.37	1.18
**%Loop**	0.09	0.05	0.08	0.05	0.06	0.04	0.07	0.08	0.06	0.05
**SWTL**	2.10	2.00	2.01	1.84	1.91	1.87	1.92	1.92	1.93	1.94
**MaxTL**	3.69	3.39	3.58	3.58	3.58	3.58	3.58	3.58	3.58	3.80
**ChLen**	1.94	1.92	1.91	1.94	1.96	1.94	1.95	1.94	1.96	1.86
**%Omn**	0.72	0.71	0.70	0.73	0.73	0.71	0.76	0.71	0.73	0.68
**%Can**	0.15	0.16	0.16	0.16	0.14	0.15	0.14	0.11	0.15	0.10
**Path**	2.05	2.03	2.06	2.05	2.10	2.09	2.07	2.15	2.10	2.31

### 4. Comparison with other aquatic food webs

We used MDS ordination to compare the structure of our cumulative regional and overall food webs (Atlantic, NB, PEI, NS; classified as temperate seagrass webs) with 14 other aquatic food webs across six ecosystem groups (marine, estuarine, lotic, lentic, seagrass-tropical and seagrass-temperate). The results showed that our seagrass webs were more similar to each other than to any other web, and the next most similar web was that of a tropical seagrass-dominated estuary, Saint Mark's estuary in Florida (Fig. 4d). PERMANOVA followed by pair-wise t-tests confirmed that our temperate seagrass webs tended to be different from all other food webs (p = 0.063), except for the tropical seagrass-dominated estuary (p = 0.19). Overall, the temperate and St Mark's estuary seagrass webs were characterized by lower fractions of %Omn and %I, a higher fraction of %T, and lower C relative to the other aquatic webs.

### 5. Extinction analysis

**Figure 8:**
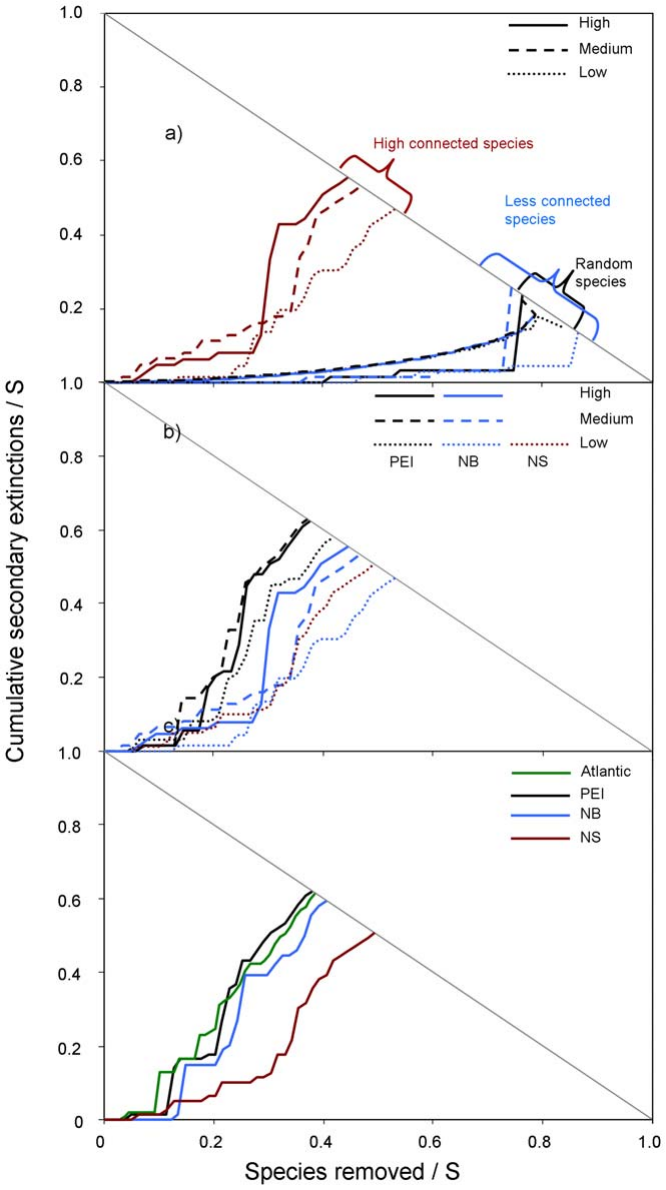
Cumulative secondary extinctions resulting from primary loss of species in food webs. A) Lost of most, least, and randomly connected species in food webs by eutrophication level from New Brunswick (NB), b) most connected species in food webs by region and eutrophication level from NB, Prince Edward Island (PEI) and Nova Scotia (NS), and c) most connected species in cumulative webs by region and the overall Atlantic seagrass web.

To test whether changes in food-web structure translated into changes in functioning, we analyzed the robustness of food webs to simulated species loss. Food webs from high impact sites were less robust to species deletion than those from medium or low eutrophication when the most connected or random species were deleted for NB and PEI (Fig. 8a, b). Overall, PEI webs were the least robust to species deletion, followed by NB and NS (Fig. 8b, c). The Atlantic web fell between the cumulative PEI and NB webs.

## Discussion

Seagrass beds provide important structure, functions, and services to coastal ecosystems, yet how these differ across different spatial scales and change with human impacts has not been rigorously quantified. We present a detailed characterization of food webs associated with seagrass beds in different regions in Atlantic Canada, and show how these food webs change across a gradient of human impacts associated with eutrophication. Whereas food-web structure was similar among low-impact sites, increasing food-web degradation was observed with rising impact level indicated by a structural simplification and lower robustness to species loss. Overall, our temperate seagrass food webs were similar to a tropical seagrass food web, yet different from other aquatic webs, suggesting that seagrass ecosystems may differ structurally from other aquatic webs. Our results also show that the spatial scale of study is an important factor for food-web analysis. These results may inform conservation criteria and future management plans of coastal areas in Atlantic Canada since they provide reference directions of degradation of temperate seagrass beds in the region.

### 1. Seagrass food webs in Atlantic Canada

Although comparable in overall topology, the temperate seagrass food webs differed from other, previously characterized, aquatic ecosystems except a tropical seagrass-dominated web from the Saint Mark's estuary in Florida [20], [27], [32]. In the marine realm, seagrass beds are among the few ecosystems that provide both habitat and a primary resource for associated organisms, and thus may be structurally different from webs that don't contain extensive beds of foundation vegetation.

Within Atlantic Canada, seagrass food webs from low impact sites in NS, NB, and PEI showed similar food-web structure suggesting consistent features across local and regional scales, despite differences in localities (such as the connection to the open Atlantic Ocean in NS, open Gulf of St. Lawrence in PEI, and Northumberland Strait in NB) and food-web composition (Fig. 2). However, we also observed changes in food-web structure with increasing human impacts in both NB and PEI, although the responses were not always consistent between the two regions (see the discussion below).

### 2. Changes in food-web structure with eutrophication

Eutrophication in seagrass webs has been shown to reduce above and below ground seagrass production, decrease shoot density, and increase the abundance of fast-growing phytoplankton, epiphytic and benthic algae [11]. These direct effects were also observed at our study sites (Table 1, Methods S1, [12], [14]). The resulting enhanced turbidity, overgrowth, shading and oxygen depletion due to enhanced decomposition can then lead to increasing canopy patchiness or, in the extreme, complete canopy loss [4], [12], [13]. All our study sites maintained seagrass canopies and thus did not represent extreme levels of eutrophication. However, eutrophication-induced changes in the composition of primary producers and canopy structure can alter associated species abundance and diversity [12], [33], [34]. Changes in species presence/absence, or local diversity, should be evident as structural changes in the food webs.

In our study, higher impact levels were associated with changes in species composition at all trophic levels (from primary producers to consumers, Methods S2), which translated into some changes in food-web structure. In PEI and NB, several food-web properties followed a trend towards higher degradation with increasing impact level, although there was considerable variability in the response of individual food-web properties. At high impact sites, food webs generally showed reduced diversity (less trophic groups) and trophic height (lower maximum trophic level of the highest top predator), and a simplification of trophic complexity (fewer number of trophic links connecting top to basal species). We also found an increase in lower-trophic level groups (higher fraction of herbivores and intermediate consumers), grazers and detritivores such as gastropods and small decapods (higher ratio of prey per species). PEI sites also showed a decrease in the fraction of top predators, lower fractions of omnivory and cannibalism, and a shorter trophic path length. These results coincide with several changes described for three food webs constructed along a gradient of eutrophication in the Montego estuary in Portugal [17], although in Montego estuary seagrass beds had disappeared at highly eutrophied sites due to the severity of impacts.

Some food-web properties did not follow our expected initial trends of degradation (Table 2). The increase in linkage density, connectance, and the ratio of predator to prey species contrasts with our initial predictions, and shows the specific structural impact of the increase in primary producers, herbivores, and intermediate species, as well as the changes in the three-dimensional structure of the seagrass beds. As eutrophication increases there is a reduction in shoot density, decreases in above and below ground biomass, and an overall reduction in the physical complexity of the habitat [12], [14]. These changes reduce the suitability of the habitat as nursery, sheltering, and foraging areas to various organisms [4], [5], [9], [10], and may increase interaction strengths of remaining species and their exposure to predation.

Several properties tended to show non-linear trends from low to high eutrophication sites (Fig. 5). This may be explained by a successional increase in biomass and secondary production due to the increase in food supply [35] or an “enrichment phase” [36]. Moreover, eutrophication impacts were not consistent between NB and PEI. In both regions chlorophyll-a levels generally increased [14] and C/N ratios in seagrass tissues decreased from low to high impact levels. However, sites in block 2 in NB showed increasing epiphytic algal biomass on seagrass blades, while block 4 in PEI had high benthic annual algal biomass, mostly *Ulva* spp. (Methods S1, [14], A. Schmidt unpublished data). These results highlight the complexity of bottom-up effects on seagrass food webs. Eutrophication can favor different primary producer groups [4], [11], [15], [37] in different coastal ecosystems, which may depend on site-specific abiotic and biotic conditions. Such differential changes in primary producers can then translate into site-specific changes in food-web structure. Understanding these changes is essential for the proper protection and management of coastal ecosystems.

### 3. Changes in food-web functioning

Differences in food-web structure due to diversity changes can also affect the robustness of communities to species loss. Overall, seagrass food webs from high impacted sites were less robust than those from medium or low impacted sites to simulated species loss, and the impact was higher when deleting the most connected species compared to less connected or random species. Thus, our results suggest that as seagrass food webs become more degraded they become more vulnerable to the loss of species that highly interact in the web. These results are comparable to previous studies simulating the loss of species [20], [32]: more secondary extinctions occur when removing highly connected species, followed by random, and less connected species. This has implications for ecosystem management since highly exploited species tend to be highly connected in marine food webs [22], [38].

Interestingly, seagrass food webs from PEI were less robust to species loss than those from NB and NS, which may be related to the overall greater degradation observed in the PEI webs. Our results showed that 75% of all properties in site-specific food webs in PEI followed the expected trend of degradation compared to only 43% in NB, however these ratios differed for cumulative webs (69% in NB, 50% in PEI).

Overall, our results suggest that food webs that are subjected to a higher degree of anthropogenic impacts are more degraded, simplified, and less robust to species extinctions, patterns that have been previously shown for marine food webs in the Mediterranean [22] and seagrass food webs in Portugal [17]. Thus the binary food-web network approach chosen in this study captures fundamental processes in the response of food webs to degradation. However, the more specific effects of changes in species abundance, biomass and energy flows in food webs, which are very important as a response to eutrophication, need to be studied with more complex modeling tools (e.g. [17]).

### 4. Multiple stressors and seagrass beds

Declines in seagrass beds have frequently been the result of a combination of anthropogenic and natural impacts [7]. In addition to nutrient loading (Table 1), our study sites may be affected by other factors, such as chemical pollution, land clearing and construction, and fisheries, as well as changes in the marine and terrestrial fauna that are using seagrass beds [12]. These multiple stressors may interact with each other, can enhance or dampen each other's effect, and challenge our understanding of eutrophication impacts on coastal ecosystems since their analysis is complex [39], [40]. For example, although separated in terms of eutrophication properties (Table 1), Cocagne (classified as medium impacted site in NB) and Bouctouche (classified as high) showed similar food-web structure (Table 3), possibly due to the combined effect of multiple human or natural stressors [12]. Higher food-web degradation in PEI may reflect the high impact of farming (mostly potato), with associated high loads of fertilizers and pesticides that partially end up in estuaries ([41], Table 1). While fertilizers directly enhance nutrient loading, pesticides can have severe effects on immune and reproductive systems and growth and production of marine biota [39]. On the other hand, recreational fisheries may be important factors at some sites as fishing for invertebrates is a common practice in Atlantic Canada [42]. Removal of herbivorous invertebrates can accelerate the impacts of eutrophication on seagrass due to removal of grazing control [37] and can influence the system-level response to nutrient enrichment [43]. All of these factors may have altered the site-specific response to eutrophication and may explain the variability we observed in our results.

### 5. The spatial scale of food-web analysis

Finally, our results illustrate the importance of spatial scale for understanding how food webs are structured and how they function. Cumulative or aggregated food webs are useful to represent and compare food-web structure of larger regions (NS, NB, PEI, Atlantic). However, these cumulative webs produce different results from food webs at smaller spatial scales (study sites, region by impact level, or region). These differences are mainly driven by changes in the number of trophic groups that occur at each site and their ecological roles, as well as by the uncertainty of the data and sampling limitations. Therefore, the best approach to study food-web structure and functioning may be to combine ecological data with different spatial resolutions. Neighboring areas may show significant differences in food-web organization driven by local or regional factors that are overlooked when data are integrated and only cumulative food webs are studied, as commonly done (e.g. [21], [38]). Similar conclusions regarding the importance of the spatial scale of study were drawn in an analysis of data collected in several streams at various spatial scales [44] and more generally in other ecosystems [45]. This is a relevant issue in food-web ecology in general as food webs are typically assembled in aggregated forms (cumulative or summary webs) due to limited data availability on trophic interactions.

## Supporting Information

Methods S1 **Study sites by region, block and eutrophication level.** Exposure conditions and mean carbon to nitrogen (C/N) ratios in seagrass tissue, annual and filamentous epiphytic (on seagrass blades) and benthic algal biomass (g/m^2^), and chlorophyll-a concentrations in the water column (µg/L) (±SE) are reported for each site.
(DOC)

Methods S2 **Species sampled in seagrass beds at each site in New Brunswick (NB), Prince Edward Island (PEI), and Nova Scotia (NS) from July - August 2007.** Presence (+) or absence (−) is shown for each site for Low/Medium/High impacted sites in each block (1–4) for NB and PEI, and for each site Taylor Head Provincial Park/False Passage/Musquodoboit Harbour/Franks George in NS (a single sign is used when records were the same in all sites).
(DOC)

Methods S3 **Trophic information from the literature used to assemble the seagrass food-web networks.**
(DOC)

Methods S4 **Trophic groups used to assemble the seagrass food-web networks.** Not all groups were used in all food webs; see Methods S2 for detailed occurrence information.
(DOC)
