# Supplementary material for: Food-Web Structure of Seagrass Communities across Different Spatial Scales and Human Impacts
Source: PLoS One. 2011 Jul 21;6(7):e22591. doi: 10.1371/journal.pone.0022591 (PMC3141067; doi:10.1371/journal.pone.0022591)
Supplement: Methods S1 — Study sites by region, block and eutrophication level. Exposure conditions and mean carbon to nitrogen (C/N) ratios in seagrass tissue, annual and filamentous epiphytic (on seagrass blades) and benthic algal biomass (g/m2), and chlorophyll-a concentrations in the water column (µg/L) (±SE) are reported for each site. (DOC) [file pone.0022591.s001.doc]

Methods S1: Study sites by region, block and eutrophication level. Exposure conditions and mean carbon to nitrogen (C/N) ratios in seagrass tissue, annual and filamentous epiphytic (on seagrass blades) and benthic algal biomass (g/m^2^), and chlorophyll-a concentrations in the water column (μg/L) (±SE) are reported for each site.

|  |  |  |  |  |  |  |  |
| --- | --- | --- | --- | --- | --- | --- | --- |
| **Code** | **Code** | **Location** | **Eutrophication** | **Chl-a (µg/l)** | **C/N** | **Epiphytic Algal Biomass (g/m^2^)** | **Benthic Algal Biomass (g/m^2^)** |
| **New Brunswick** |  |  |  |  |  |  |  |
| **Block 1** |  |  |  |  |  |  |  |
| TB | TB | N 47°22’56, W 64°56’21 | Low | 3.43 (0.93) | 31.8 (2.3) | 0.07 (0.07) | 0 (0) |
| BS | BS | N 46° 29’70, W 64°40’47 | Medium | 4.54 (1.37) | 30.9 (1.7) | 0 (0) | 0 (0) |
| LM | LM | N 47°47’44, W 64°40’31 | High | 11.3 (4.38) | 24.5 (0.7) | 4.2 (2.9) | 0 (0) |
| **Block 2** |  |  |  |  |  |  |  |
| KB | KB | N 46° 50’30, W 64°56’16 | Low | 3.43 (1.07) | 28.4 (0.5) | 0 (0) | 0 (0) |
| CG | CG | N 46°22’01, W 64°36’95 | Medium | 8.28 (3.47) | 23.4 (0.4) | 190.2 (49.2) | 0 (0) |
| BT | BT | N46°29’70, W 64°47’47 | High | 11.7 (2.55) | 23.1 (0.5) | 563.1 (229.4) | 0.933 (0.933) |
| **Prince Edward Island** |  |  |  |  |  |  |  |
| **Block 3** |  |  |  |  |  |  |  |
| ST | ST | N 46°28’47, W 63°27’84 | Low | 9.12 (1.13) | 38.5 (1.5) | 16.4 (8.6) | 2.1 (2.1) |
| MD | MD | N 46°25’01, W 62°37’60 | Medium | 6.72 (0.32) | 25.7 (0.02) | 43.6 (30.7) | 612.5 (417.7) |
| SW | SW | N 46°28’75, W 63°30’38 | High | 12.6 (2.51) | 22.7 (2.1) | 74.5 (64.6) | 1012.4 (568.4) |
| **Block 4** |  |  |  |  |  |  |  |
| FL | FL | N 46°41’29, W 63°56’40 | Low | 3.26 (0.69) | 30.3 (2.5) | 1.15 (1.15) | 0.53 (0.53) |
| MR | MR | N 46°45’91, W 64°04’72 | Medium | 11.3 (0.41) | 32.1 (2.1) | 18.4 (10.8) | 7.33 (7.33) |
| KD | KD | N 46°49’96, W 64°02’97 | High | 12.3 (2.60) | 19.8 (-) | 0 (0) | 3068.4 (1534.1) |
| **Nova Scotia** |  |  |  |  |  |  |  |
| TH | TH | N 44°49’26, W 62°34’32 | Low | 1.82 (0.27) | 28.2 (0.7) | 31.9 (29.3) | 5.1 (2.3) |
| FP | FP | N 44°44’37, W 62°47’45 | Low | 1.16 (0.07) | 31.3 (2.8) | 30.0 (30.2) | 25.5 (13.2)^1^ |
| MH | MH | N 44°42’46, W 63°04’48 | Low | 5.72 (0.97) | 23.2 (2.2) | 3.52 (3.52) | 0.29 (0.29) |
| FG | FG | N 44°35’68, W 63°53’73 | Low | 1.27 (0.08) | 31.0 (1.5) | 21.4 (16.4) | 111.7 (82.0)^2^ |
| 1- mainly *Ceramium* *rubrum* and *Polysiphonia* sp.; 2 - mainly *Sphaerotrichia divaricata.* | | | | | | | |
