# Supplementary material for: Food-Web Structure of Seagrass Communities across Different Spatial Scales and Human Impacts
Source: PLoS One. 2011 Jul 21;6(7):e22591. doi: 10.1371/journal.pone.0022591 (PMC3141067; doi:10.1371/journal.pone.0022591)
Supplement: Methods S2 — Species sampled in seagrass beds at each site in New Brunswick (NB), Prince Edward Island (PEI), and Nova Scotia (NS) from July - August 2007. Presence (+) or absence (−) is shown for each site for Low/Medium/High impacted sites in each block (1–4) for NB and PEI, and for each site Taylor Head Provincial Park/False Passage/Musquodoboit Harbour/Franks George in NS (a single sign is used when records were the same in all sites). (DOC) [file pone.0022591.s002.doc]

Methods S2: Species sampled in seagrass beds at each site in New Brunswick (NB), Prince Edward Island (PEI), and Nova Scotia (NS) from July - August 2007. Presence (+) or absence (-) is shown for each site for Low/Medium/High impacted sites in each block (1-4) for NB and PEI, and for each site Taylor Head Provincial Park/False Passage/Musquodoboit Harbour/Franks George in NS (a single sign is used when records were the same in all sites).
