# Supplementary material for: Food-Web Structure of Seagrass Communities across Different Spatial Scales and Human Impacts
Source: PLoS One. 2011 Jul 21;6(7):e22591. doi: 10.1371/journal.pone.0022591 (PMC3141067; doi:10.1371/journal.pone.0022591)
Supplement: Methods S3 — Trophic information from the literature used to assemble the seagrass food-web networks. (DOC) [file pone.0022591.s003.doc]

Methods S3: Trophic information from the literature used to assemble the seagrass food-web models.
