# Supplementary material for: Food-Web Structure of Seagrass Communities across Different Spatial Scales and Human Impacts
Source: PLoS One. 2011 Jul 21;6(7):e22591. doi: 10.1371/journal.pone.0022591 (PMC3141067; doi:10.1371/journal.pone.0022591)
Supplement: Methods S4 — Trophic groups used to assemble the seagrass food-web networks. Not all groups were used in all food webs; see Methods S2 for detailed occurrence information. (DOC) [file pone.0022591.s004.doc]

Methods S4: Functional groups used to assemble the seagrass food-web models. Not all groups were used in all food webs; see Methods S2 for detailed occurrence information.
